# Research Status and Emerging Trends in Virtual Reality Rehabilitation: Bibliometric and Knowledge Graph Study

**DOI:** 10.2196/41091

**Published:** 2023-03-06

**Authors:** Ting Fan, Xiaobei Wang, Xiaoxi Song, Gang Zhao, Zhichang Zhang

**Affiliations:** 1 Department of Computer School of Intelligent Medicine China Medical University Shenyang China; 2 Department of General Practice The First Hospital of China Medical University China Medical University Shenyang China; 3 Liaoning Education Informatization Construction Center LiaoNing Institute of Education Shenyang China; 4 Department of Health Promotion School of Intelligent Medicine China Medical University Shenyang China

**Keywords:** mobility, rehabilitation, virtual reality, bibliometric, technology, training, interactive, research, exercise, resources, cerebral palsy, adult, video games

## Abstract

**Background:**

Virtual reality (VR) technology has been widely used in rehabilitation training because of its immersive, interactive, and imaginative features. A comprehensive bibliometric review is required to help researchers focus on future directions based on the new deﬁnitions of VR technologies in rehabilitation, which reveal new situations and requirements.

**Objective:**

Herein, we aimed to summarize effective research methods for and potential innovative approaches to VR rehabilitation by evaluating publications from various countries to encourage research on efficient strategies to improve VR rehabilitation.

**Methods:**

The SCIE (Science Citation Index Expanded) database was searched on January 20, 2022, for publications related to the application of VR technology in rehabilitation research. We found 1617 papers, and we created a clustered network, using the 46,116 references cited in the papers. CiteSpace V (Drexel University) and VOSviewer (Leiden University) were used to identify countries, institutions, journals, keywords, cocited references, and research hot spots.

**Results:**

A total of 63 countries and 1921 institutes have contributed publications. The United States of America has taken the leading position in this field; it has the highest number of publications; the highest h-index; and the largest collaborative network, which includes other countries. The reference clusters of SCIE papers were divided into the following nine categories: kinematics, neurorehabilitation, brain injury, exergames, aging, motor rehabilitation, mobility, cerebral palsy, and exercise intensity. The research frontiers were represented by the following keywords: *video games* (2017-2021), and *young adults* (2018-2021).

**Conclusions:**

Our study comprehensively assesses the current research state of VR rehabilitation and analyzes the current research hot spots and future trends in the field, with the aims of providing resources for more intensive investigation and encouraging more researchers to further develop VR rehabilitation.

## Introduction

In recent years, the number of people with rehabilitation needs has increased, particularly among groups of older patients, patients with disabilities, patients with chronic diseases, and patients with functional and cognitive impairments. The loss of movement, sensation, balance, and cognition, as well as other aspects, seriously affects patients’ quality of life, work, study, and social life [1,2]. Such patients require long-term, consistent rehabilitation training and guidance [3]. However, traditional rehabilitation training has a number of problems, including fixed rehabilitation centers, a lack of rehabilitation resources, uninteresting training processes, high treatment costs, and a lack of automatic guidance and incentive mechanisms. These result in a lack of confidence in the rehabilitation process, which in turn affects the outcomes of rehabilitation treatments [4,5].

With the gradual popularization of virtual reality (VR) technology, rehabilitation training systems based on VR technology have been gradually applied in sports, exercise, and functional rehabilitation for various diseases and have achieved positive effect results [6,7]. The combination of VR technology and rehabilitation medicine can enable more patients to train regularly at home or in the community, as VR rehabilitation systems provide an immersive experience that stimulates patients’ interest and improves their participation, thus overcoming the disadvantages of fixed centers and the lack of resources [8]. Furthermore, VR rehabilitation systems can sense and record a patient’s movement and biological data via sensors to further improve existing rehabilitation programs [9]. This rehabilitation technology is a useful supplement to traditional rehabilitation and is a promising new research direction in the field of rehabilitation medicine.

A comprehensive bibliometric review is required to help researchers focus on future directions based on the new deﬁnitions of VR technologies in rehabilitation, which reveal new situations and requirements. Although bibliometric methods have yielded positive results in a variety of ﬁelds, we found that there is still a significant gap in the research on VR rehabilitation and its development trends by using bibliometric methods.

We used bibliometric methods to analyze SCIE (Science Citation Index Expanded) papers on studies related to VR rehabilitation research. Articles from different countries, regions, and research institutions were included. We identified papers in journals, gathered the top 10 citations, and enumerated how many times these citations were used. The VR rehabilitation knowledge base was analyzed by grouping authors’ co-occurring keyword networks. Burst citations were used to identify research hot spots on this topic, which could provide a useful reference for future research [10,11]. These analyses will provide rehabilitation specialists with a macroscopic understanding of the knowledge domain as a whole, as well as a microscopic characterization. Compared to other reviews, our study is timely and visual and provides an impartial approach to developing and exploring particular knowledge domains. Our findings may encourage more researchers to conduct additional research in this field to further develop VR rehabilitation methods. The following basic information was gathered from studies: titles, abstracts, author information, institutions, countries, regions, keywords, and citations.

## Methods

**Figure 1:**
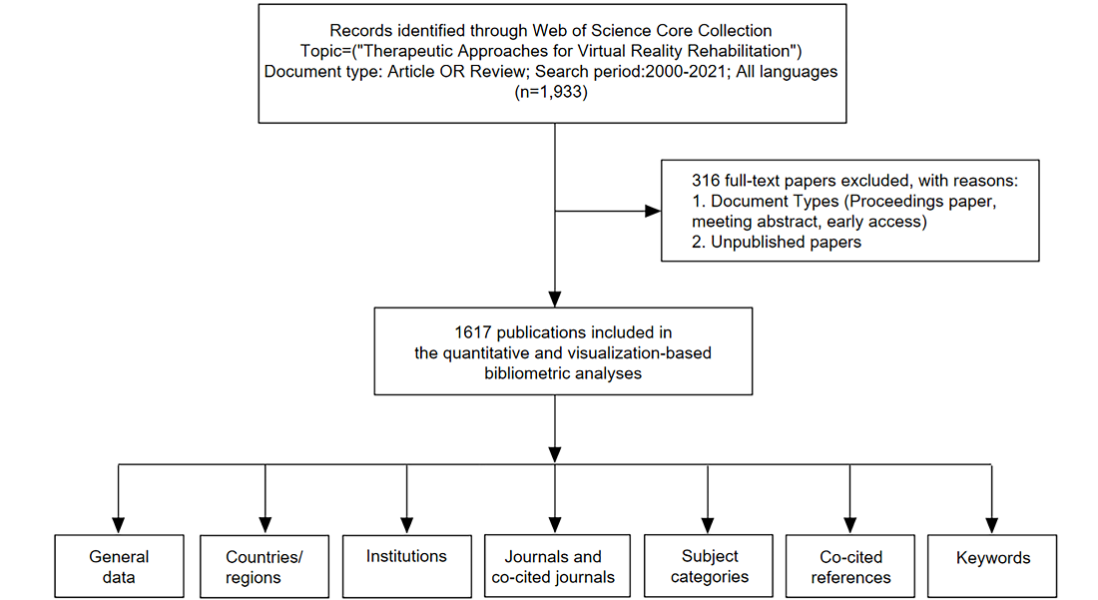
PRISMA (Preferred Reporting Items for Systematic Reviews and Meta-Analyses) flow diagram of study selection. The diagram shows details on the selection criteria for virtual reality rehabilitation publications from the SCIE (Science Citation Index Expanded) database and the steps of bibliometric analysis.

We created a clustered network, using the 46,116 references cited in 1617 published papers. The data came from the Web of Science Core Collection (WSCC), which was accessed on January 20, 2022. The search covered articles published from 2000 to 2021. The following search string, which included the search terms, was used: *Virtual Reality OR VR OR AR OR Augmented Reality*. Further, we limited our search to original articles and reviews, and the Web of Science category *Rehabilitation* was selected. To determine study inclusion, the aforementioned basic information was collected from articles in the WSCC. However, the following papers were excluded: (1) irrelevant proceedings and meetings papers, (2) chapters in books, (3) duplicated articles, and (4) unpublished papers with limited information. In total, 1617 papers were included, with duplicates excluded. The search and analysis procedures are outlined in Figure 1.

We defined most publication traits, including institute, country, journal, and keywords. We used Journal Citation Reports (2020 version; Clarivate) to identify impact factors, which reflect scientific research value [12]. All data were processed by using CiteSpace V (Drexel University) and VOSviewer (Leiden University). Both programs are used for collaboration network analysis to link publication traits [11,13]. From these analyses and measures, we obtained data on research hot spots, evolution paths, knowledge structures, and new trends in VR rehabilitation.

## Results

### Study Distribution by Publication Year

**Figure 2:**
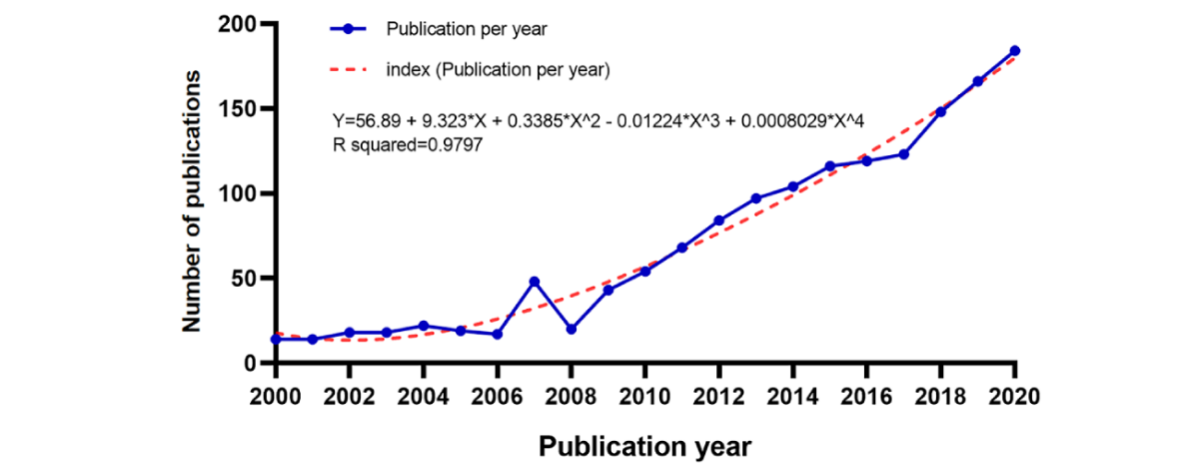
Trends in the number of publications on virtual reality rehabilitation.

Between 2000 and 2021, a total of 1617 papers on VR rehabilitation were published. Emerging trends in VR rehabilitation research–related studies are outlined in Figure 2. From 2000 onward, VR rehabilitation research rapidly accelerated. In Figure 2, the blue line denotes the increasing trend in the annual number of studies from 2000 to 2021, whereas the red broken line represents the publication index, which also increased. In the initial 2000 to 2006 research period, publication numbers per year were relatively stable and were <25, suggesting an initial period of exploratory VR rehabilitation research. However, the rise in rehabilitation demands and the increased development of supporting technologies, which are due to the increased interest in applying VR to medicine and rehabilitation and the increasing, widespread use of VR technology, have resulted in a proliferation of publications since then. The number of publications in last decade accounted for more than 80% of the total publications found.

### Analysis of Countries and Institutes

**Figure 3:**
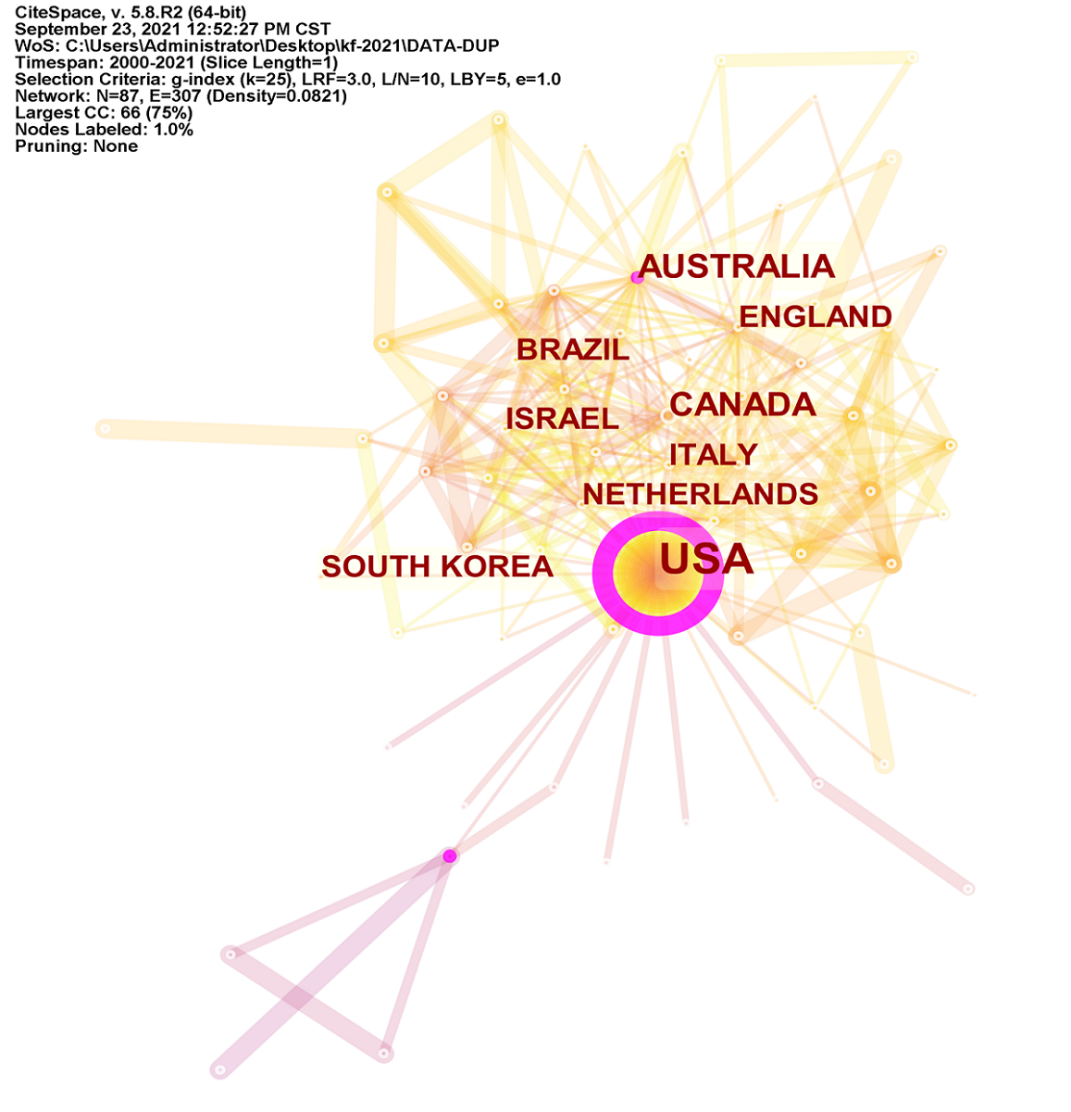
The cooperation of countries and regions that contributed to publications on virtual reality rehabilitation. CC: co-citations; CST: Central Standard Time; LBY: Look back year; L/N: Maximum Links Per Node; LRF: Link Retaining Factor; WoS: Web of Science.

**Figure 4:**
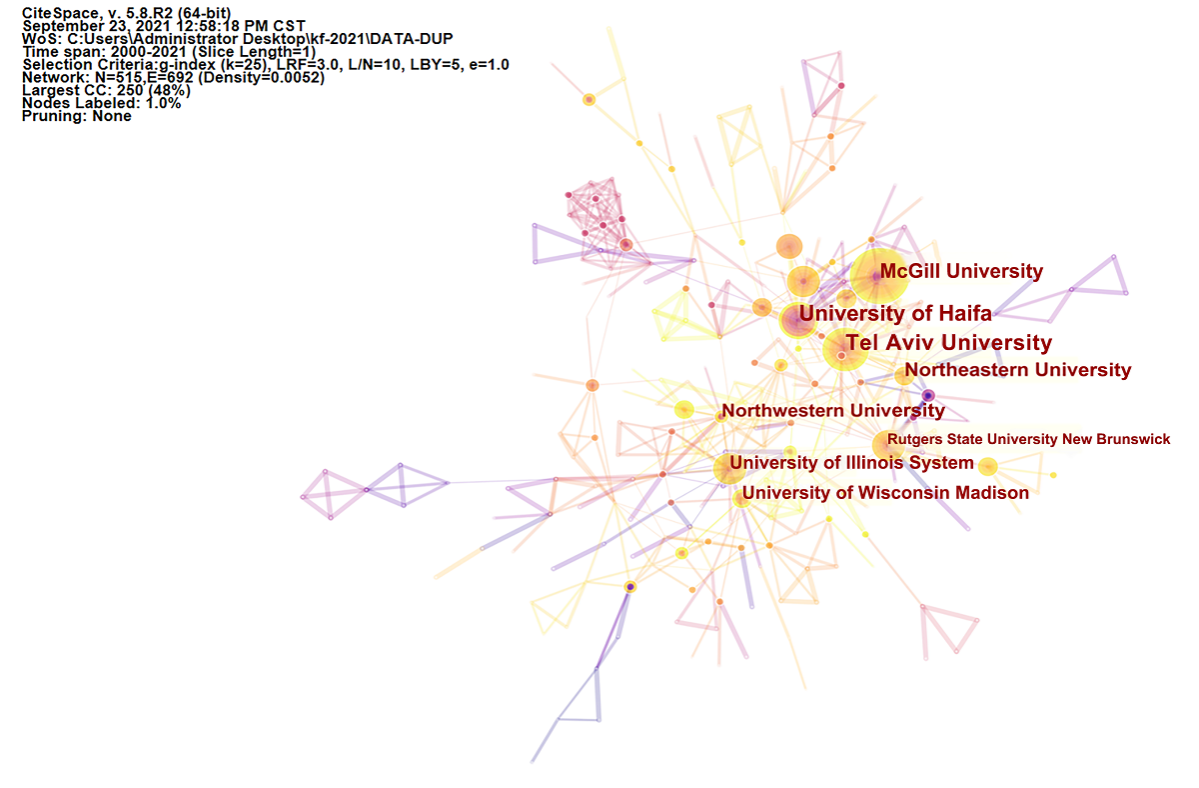
The cooperation of institutions that contributed to publications on virtual reality rehabilitation therapy. CC: co-citations; CST: Central Standard Time; LBY: Look back year; L/N: Maximum Links Per Node; LRF: Link Retaining Factor; WoS: Web of Science.

The analyzed publications included contributions from a total of 63 countries and 1921 institutes. The top 10 countries and institutes are outlined in Tables 1 and 2, respectively, whereas collaborations between countries and between institutes are shown in Figure 3 and Figure 4, respectively. The United States published the most studies (n=663), followed by Canada (n=143). The United States also had the top h-index (59), followed by Canada (29) and Australia (21). Rutgers State University New Brunswick had the most publications (n=52), followed by McGill University (n=45). Rutgers State University New Brunswick also had the highest h-index (27). On a global level, research institutes and associated research staff are collaborating and sharing experiences. However, although the United States has played a leading role, the international collaboration rates of the United States are low.

**Table 1:** The top 10 countries in terms of publications.

Rank	Countries	Publication count, n	h-index
1	United States of America	663	59
2	Canada	143	29
3	Australia	96	21
4	England	92	29
5	South Korea	83	26
6	Italy	81	26
7	Israel	71	27
8	Brazil	68	21
9	Netherlands	67	17
10	Spain	61	21

**Table 2:** Top 10 institutions in terms of publications.

Rank	Institutions	Publication count, n	h-index
1	Rutgers State University New Brunswick	52	27
2	McGill University	45	17
3	Pennsylvania Commonwealth System of Higher Education	38	13
4	Tel Aviv University	38	16
5	University of Wisconsin System	38	12
6	University of Haifa	37	19
7	University of Illinois System	35	15
8	US Department of Veterans Affairs	34	13
9	Veterans Health Administration	34	13
10	University of Wisconsin Madison	33	11

### Analysis of Journals

**Figure 5:**
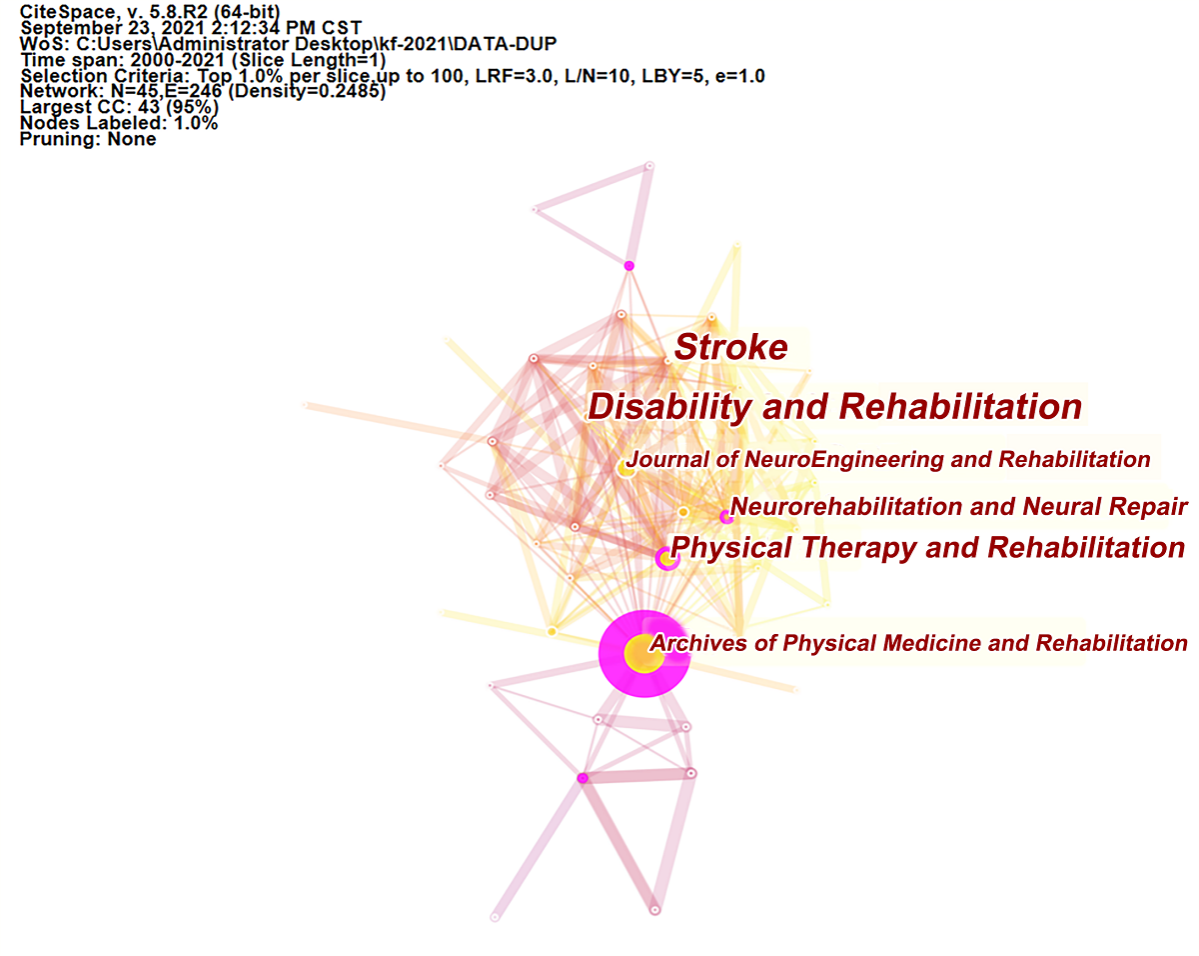
The network map of cited journals contributed to publications on virtual reality rehabilitation. CC: co-citations; CST: Central Standard Time; LBY: Look back year; L/N: Maximum Links Per Node; LRF: Link Retaining Factor; WoS: Web of Science.

A total of 241 journals published articles related to VR rehabilitation; the top 10 are outlined in Table 3, and collaborations between cited journals are shown in Figure 5. *Archives of Physical Medicine and Rehabilitation* was the top journal (publications: n=847), followed by *Physical Therapy and Rehabilitation* (publications: n=608). Among these journals, *Cochrane Database of Systematic Reviews* had the highest impact factor (9.266), followed by *Stroke* (7.914).

**Figure 6:**
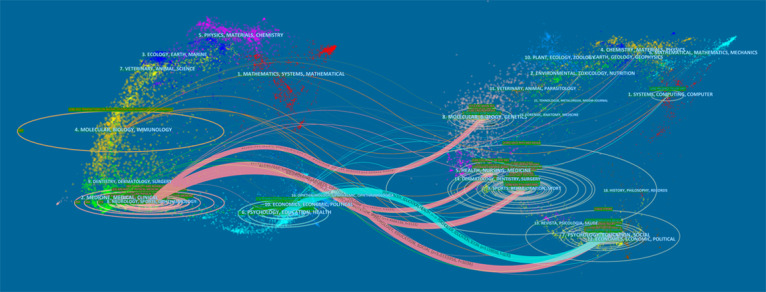
The dual map overlay of journals that contributed to publications on virtual reality rehabilitation.

Academic journals represent and reflect knowledge exchange in the research arena, where citing papers form a knowledge frontier and cited papers form a knowledge basis. A journal overlay dual map was assembled, as presented in Figure 6, wherein citing journals are shown on the left, cited journals are shown on the right, and citation relationships are represented by colored lines. These lines show that studies published in the molecular biology and genetics sector; the health, nursing, and medicine sector; the sports rehabilitation and sports sector; and the psychology, education, and social sector were usually cited in studies published in the medicine, medical, and clinical sector and the psychology, education, and health sector.

**Table 3:** The top 10 journals in terms of publications.

Rank	Cited journals	Publication count, n	h-index	Impact factor (2020)
1	*Archives of Physical Medicine and Rehabilitation*	847	197	3.966
2	*Physical Therapy and Rehabilitation*	608	150	3.021
3	*Disability and Rehabilitation*	550	111	3.033
4	*Journal of NeuroEngineering and Rehabilitation*	550	94	4.262
5	*Stroke*	484	319	7.914
6	*Neurorehabilitation and Neural Repair*	454	106	3.919
7	*CyberPsychology & Behavior*	420	143	4.157
8	*Clinical Rehabilitation*	411	110	3.477
9	*Journal of Rehabilitation Medicine*	357	96	2.912
10	*Cochrane Database of Systematic Reviews*	324	273	9.266

### Analyzing Citations

A citation is a vital bibliometric indicator, with frequently cited studies greatly inﬂuencing their research areas. We list the top 10 most highly cited publications in Table 4. *Effectiveness of Virtual Reality Using Wii Gaming Technology in Stroke Rehabilitation* by Saposnik et al [14], which was published on 2010 in *Stroke*, was the most highly cited article (cited 58 times); the article verified the effectiveness of rehabilitation for participants with stroke by using Wii (Nintendo Co, Ltd) gaming technology. Laver et al [15,16] published 2 articles with the same title—*Virtual reality for stroke rehabilitation*—on 2011 [15] and 2015 [16] in *Cochrane Database of Systematic Reviews*. These studies evaluated the rehabilitation impact of VR technology for patients with stroke in accordance with the status quo in different periods.

In network research, betweenness centrality reflects node importance in a network; thus, a higher betweenness centrality signifies an important study [23]. The betweenness centralities of the top 10 studies are shown in Table 4.

**Figure 7:**
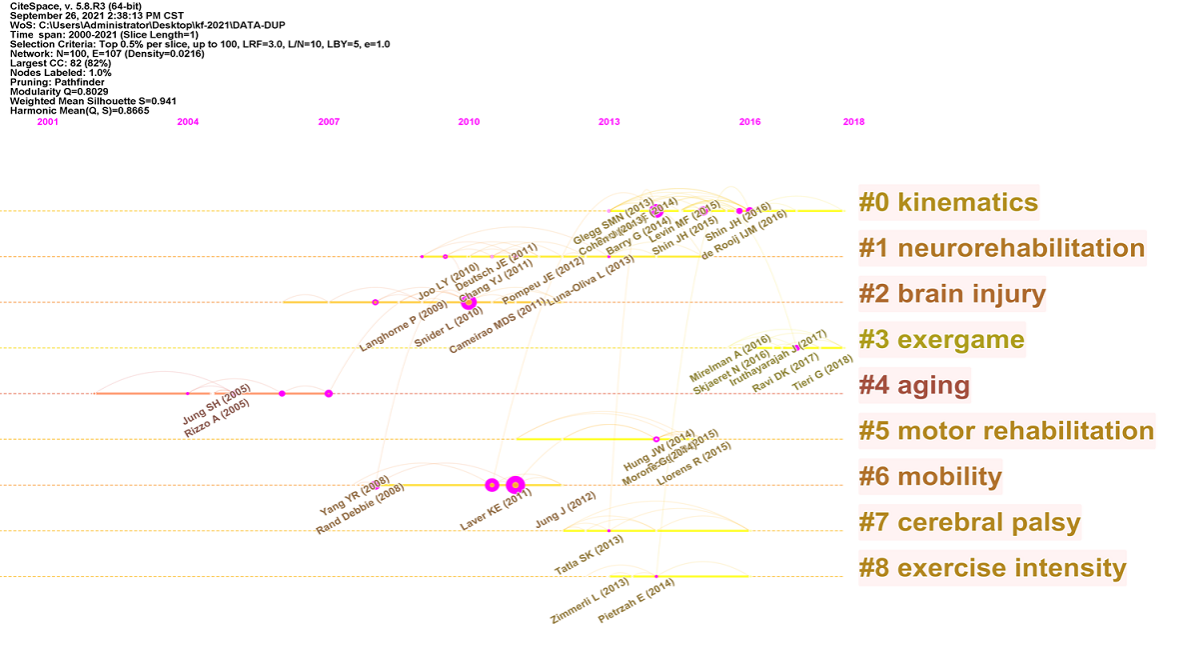
Reference cocitation map of publications on virtual reality rehabilitation. CC: co-citations; CST: Central Standard Time; LBY: Look back year; L/N: Maximum Links Per Node; LRF: Link Retaining Factor; WoS: Web of Science.

A cocited document–centered clustering investigation was proposed to identify subnodes and connecting nodes in VR rehabilitation. To assess the scientific relevance of publications, we established a network of cocited references (Figure 7). The cluster settings were as follows: the number of years per slice was set to 1, and the top 0.5% of articles were selected for analysis; a pruning algorithm was used. The modularity Q score was 0.8029 (>0.5), which showed that the network was well separated into loosely coupled clusters. The weighted mean silhouette score was 0.941 (>0.5) and suggested acceptable cluster homogeneity. Study index items were used as cluster markers. The largest cluster—cluster 0—was labeled as *kinematics*, cluster 1 was labeled as *neurorehabilitation*, cluster 2 was labeled as *brain injury,* cluster 3 was labeled as *exergame*, cluster 4 was labeled as *aging*, cluster 5 was labeled as *motor rehabilitation*, cluster 6 was labeled as *mobility*, cluster 7 was labeled as *cerebral palsy*, and cluster 8 was labeled as *exercise intensity*.

**Table 4:** The top 10 cited articles on virtual reality (VR) in rehabilitation.

Rank	Authors and published year	Title of cited article	Citation count, n	Centrality	Interpretation of the findings
1	Saposnik et al [14], 2010	*Effectiveness of Virtual Reality Using Wii Gaming Technology in Stroke Rehabilitation*	58	0.04	This article verified the effectiveness of rehabilitation for participants with stroke by using Wii (Nintendo Co, Ltd) gaming technology.
2	Saposnik and Levin [17], 2011	*Virtual Reality in Stroke Rehabilitation: A Meta-Analysis and Implications for Clinicians*	51	0.10	This study performed a meta-analysis to determine the added benefit of VR technology in arm motor recovery after stroke.
3	Deutsch et al [18], 2008	*Use of a Low-Cost, Commercially Available Gaming Console (Wii) for Rehabilitation of an Adolescent With Cerebral Palsy*	37	0.04	This document is the first report on using low-cost, commercially available gaming technology for the rehabilitation of an adolescent with cerebral palsy.
4	Laver et al [15], 2011	*Virtual reality for stroke rehabilitation*	37	0.12	This study evaluated the effects of VR and interactive video gaming on upper limb, lower limb, and global motor function.
5	Lohse et al [6], 2014	*Virtual Reality Therapy for Adults Post-Stroke: A Systematic Review and Meta-Analysis Exploring Virtual Environments and Commercial Games in Therapy*	31	0.14	This analysis systematically reviewed the evidence for VR therapy in adults after stroke.
6	Levin et al [19], 2015	*Emergence of Virtual Reality as a Tool for Upper Limb Rehabilitation: Incorporation of Motor Control and Motor Learning Principles*	27	0.01	This article discussed how to exploit VR training environments and provided evidence concerning applications for upper limb motor recovery.
7	Laver et al [16], 2015	*Virtual reality for stroke rehabilitation*	27	0.02	This paper assessed the effectiveness of VR rehabilitation training.
8	Joo et al [20], 2010	*A feasibility study using interactive commercial off-the-shelf computer gaming in upper limb rehabilitation in patients after stroke*	26	0.02	The aim of this study was to assess the feasibility of using the Nintendo Wii as an adjunct to conventional rehabilitation for patients.
9	Adamovich et al [21], 2009	*Sensorimotor training in virtual reality: A review*	25	0.01	This paper discussed possible underlying mechanisms in the area of VR rehabilitation and provided some development direction.
10	Holden [22], 2005	*Virtual Environments for Motor Rehabilitation: Review*	25	0.05	In this paper, the state of VR applications in the field of motor rehabilitation was reviewed.

### Analysis of Co-occurrence and Burst Keywords

**Figure 8:**
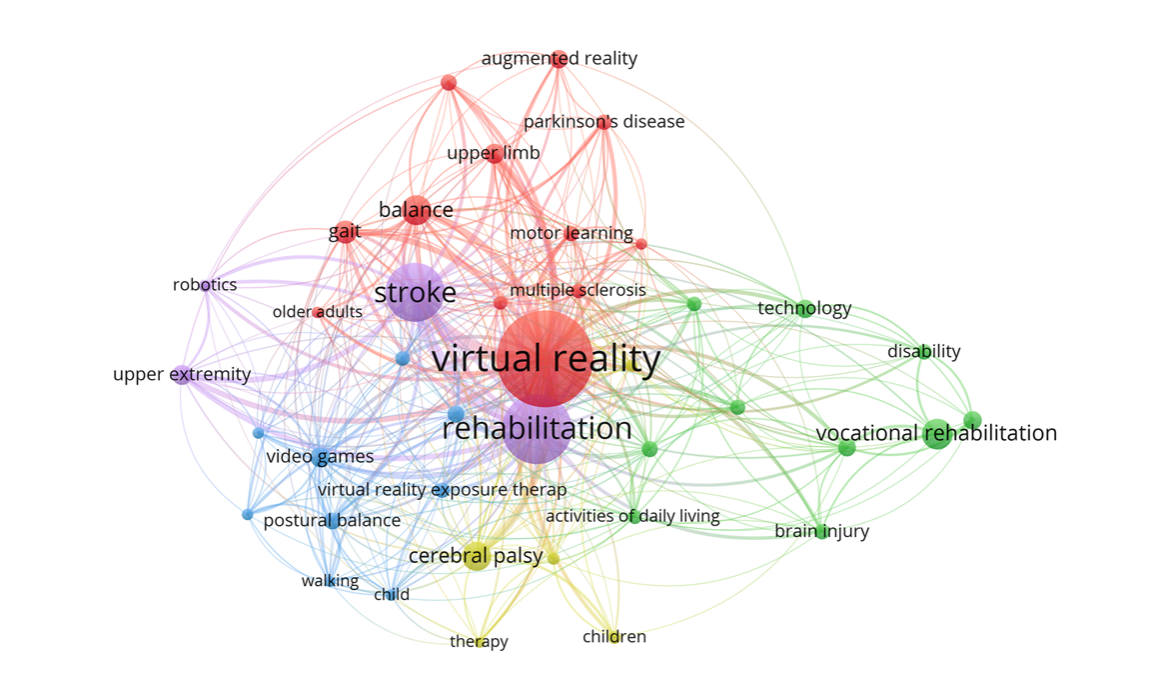
The network map of keywords. This shows 50 keywords and is divided into 9 clusters.

Keywords in similar publications were identified and processed. The top 20 keywords, which had a high link strength, are outlined in Table 5. As well as identifying thematic areas in this research field, our keyword examination of all articles (N=1617) identified 50 keywords with at least 16 occurrences (Figure 8).

**Figure 9:**
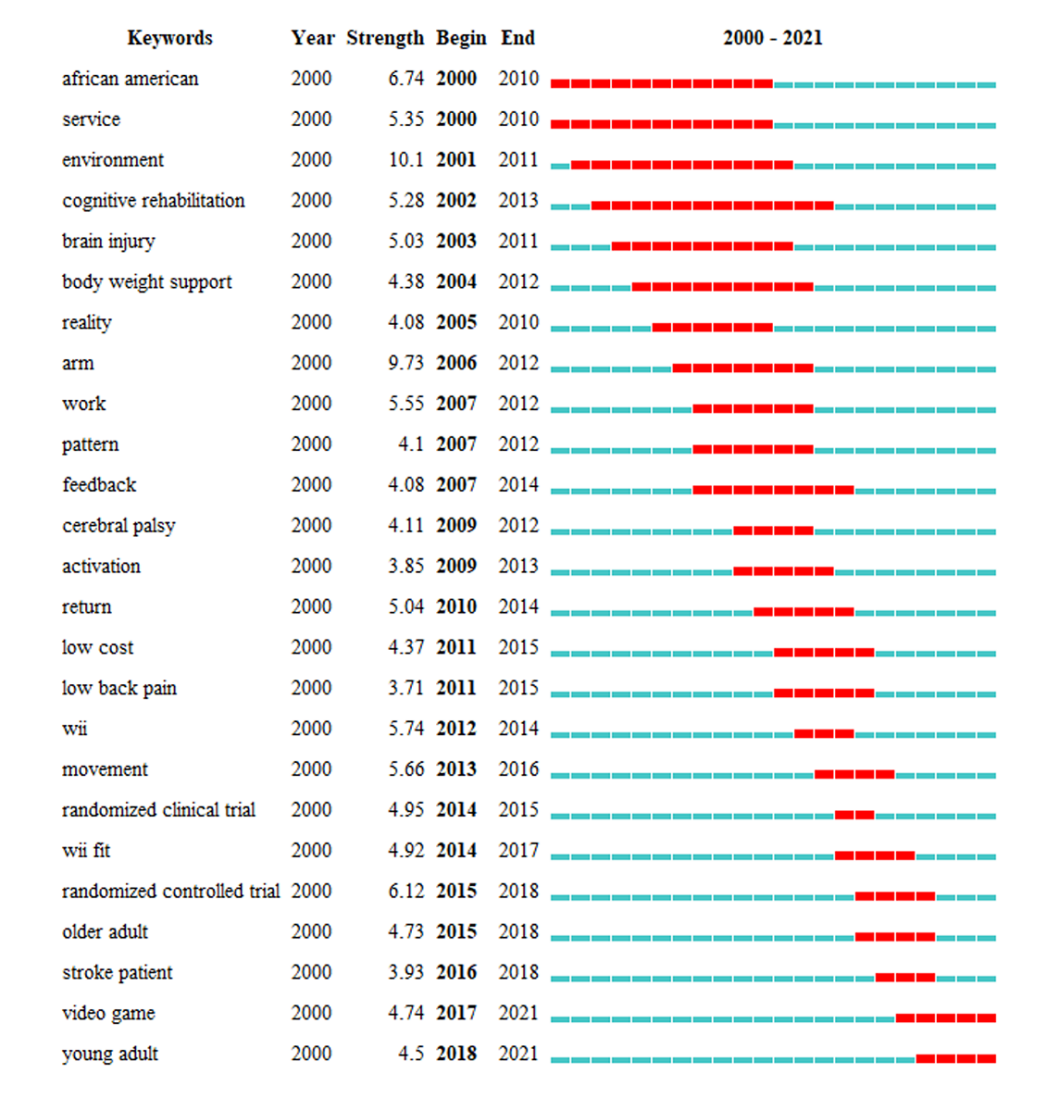
The top 25 keywords with the strongest citation bursts from publications on virtual reality rehabilitation.

We investigated hot spot shifts from a temporal perspective, using the top 2 keywords with the strongest citation burst—*video games* (2017-2021) and *young adults* (2018-2021; Figure 9).

**Table 5:** Total link strength of the top 20 occurring keywords.

Rank	Keywords	Occurrences, n	Total link strength
1	*virtual reality*	543	1305
2	*rehabilitation*	318	924
3	*stroke*	242	671
4	*vocational rehabilitation*	85	115
5	*balance*	82	232
6	*cerebral palsy*	78	205
7	*gait*	54	160
8	*upper limb*	42	138
9	*upper extremity*	41	143
10	*video games*	41	138
11	*employment*	37	80
12	*technology*	37	75
13	*augmented reality*	37	69
14	*traumatic brain injury*	35	94
15	*exercise*	32	98
16	*postural balance*	31	96
17	*telerehabilitation*	29	84
18	*cognition*	29	82
19	*disability*	28	52
20	*motor learning*	27	96

## Discussion

### General Data

This study analyzed 1617 SCIE papers that were published between 2000 and 2021 and were related to the research of VR rehabilitation. The United States published the highest number of studies (663/1617, 41.01%), and Canada published the second highest number of studies (143/1617, 8.84%). Most of the core research institutions in this field are from the United States and Israel. The US Food and Drug Administration's encouragement of digital therapy innovation is the main reason that the largest number of publications is in North American countries, led by the United States. Israel encourages entrepreneurship in the high-tech industry; as such, 4 of the top 10 universities in this field are in Israel. The most widely read journal was *Journal of NeuroEngineering and Rehabilitation*. These findings showed that *Journal of NeuroEngineering and Rehabilitation* significantly contributed to research in this area. *Journal of NeuroEngineering and Rehabilitation* focuses on the publication of research results in the fields of neuroscience, biomedical engineering, and rehabilitation, which is very much in line with the publication of papers in this field and has attracted the attention of relevant researchers. We also examined the top 10 cited publications; the top article by Saposnik et al [14] appeared in *Stroke* and was cited 58 times.

### The Knowledge Base and Current Research Characteristics

In previous studies, different VR-assisted rehabilitation systems were investigated in this field, with remarkable results. As indicated in Figure 7, after cocited reference clustering, key clustering nodes identified knowledge bases in this area, as follows: cluster 0 (*kinematics*), cluster 1 (*neurorehabilitation*), cluster 2 (*brain injury*), cluster 3 (*exergame*), cluster 4 (*aging*), cluster 5 (*motor rehabilitation*), cluster 6 (*mobility*), cluster 7 (*cerebral palsy*), and cluster 8 (*exercise intensity*). To this end, we described VR rehabilitation research knowledge bases by using different clusters, with time considerations.

For cluster 0, the *kinematics* cluster, scientists designed a VR rehabilitation system based on kinematics theory to measure, monitor, and predict outcomes; they achieved good results. For example, researchers developed various VR scenes for rehabilitation training for cervical vertebrae injuries; lower limb injuries of athletes; and arm, hand, and trunk movements in patients with stroke. They proposed kinematic evaluation and measurement methods based on the VR environment [24-26].

In cluster 1, the *neurorehabilitation* cluster, the research interest in VR technology for neurorehabilitation is increasing. Scientists have experimentally demonstrated the role of VR systems in neural rehabilitation and motor assistance from the perspectives of patients with impaired visuospatial perception after stroke, the augmented effect of intermittent theta burst stimulation on neurorehabilitation programs, and the use of motor imagination in brain-computer interfaces. Therefore, VR programs are considered safe and can be performed with standard neurorehabilitation protocols in patients with neurological conditions [27-29].

In cluster 2, the *brain injury* cluster, scientists demonstrated the experimental results of VR training systems for patients with brain injury, for those with acute onset central nervous system damage, and for vocational rehabilitation training from both the experimental perspective and the retrospective perspective. Importantly, most patients were responsive to VR training and showed improvements in gait function, balance control, vocational rehabilitation training, and other aspects [30-32].

In cluster 3, the *exergame* cluster, McMahon et al [33] investigated VR exergaming to augment physical activity in students (high school) with intellectual and developmental disabilities. Their findings indicated that when students used the VR exercise exergaming intervention, they increased the duration and intensity of their physical activity. Nambi et al [34], in their psychological and hormone analysis study, discovered that VR exergame training was effective for American soccer players with chronic low back pain when compared with conventional exercise training programs. In a multicenter controlled trial, Meyns et al [35] demonstrated that exergaming in children with spastic cerebral palsy improved balance after previous poor balance performances.

In cluster 4, the *aging* cluster, several investigations concentrated on upper limb rehabilitation intervention and overall skill improvement for older patients. Molina et al [36] analyzed the impacts of 13 VR sports games on physical function in older patients. Another group investigated the elbow and shoulder movements of older patients with chronic stroke, using the Predict Recovery Potential algorithm. The training improved motivation, but the benefits of physical function in aging were unknown [2,37].

In cluster 5, the *motor rehabilitation* cluster, scientists investigated various VR training devices. A team, for example, used VR-enhanced robot-assisted gait training to monitor the gait, motion, balance, fear of falling, and independence of 15 patients with chronic stroke. The researchers observed that cognitive performance and gait speed in tasks improved among study participants (*P*<.05) [38]. Maier at al [39], in their systematic investigation on randomized controlled trials, identified the benefits of specific VR systems for rehabilitating upper limb function and activity after stroke and reported that specific VR systems were highly beneficial for upper limb recovery when compared with conventional therapies.

In cluster 6, the *mobility* cluster, researchers designed remote VR and augmented reality rehabilitation training treadmills to record training data from patients with Parkinson disease or stroke. The experimental results showed that flexibility and balance improved and that VR rehabilitation methods could provide personalized rehabilitation strategies while also improving patient participation [40,41]. To identify the benefits of a VR-based interventional therapy, a previous scoping review reported that dynamic balance measures improved significantly following a therapeutic intervention; therefore, robust study designs that focus on intensity and dose responses from VR training could improve the efficacy of methods for treating mobility disorders [42].

In cluster 7, the *cerebral palsy* cluster, scientists studied gait, spatial perception exercises, functional mobility, exoskeletons, visuomotor construction, and other aspects of cerebral palsy in children by using data obtained through a VR rehabilitation training system. Bimanual performance and cognitive rehabilitation gains improved significantly during gait rehabilitation [43-45]. Furthermore, an augmented reality real-time feedback approach that incorporated infrared recognition technology was used to assess temporal and gait parameters during gait training in children with cerebral palsy. Importantly, cadence, velocity, functional ambulation, and bilateral step and stride length were augmented after the intervention [46].

For cluster 8, the *exercise intensity* cluster, scientists discovered that training factors (training intensity, prolonged activity, and rest were optimized) could potentially provide training optimization stimuli. de Vries et al [47] identified some elements of VR balance games that could potentially provide strength training stimulation. Baniña et al [48] investigated the relationship between upper limb motor recovery and exercise intensity in participants with subacute stroke. Importantly, they found that VR rehabilitation could be used to generate intensive exercise programs; however, clearer exercise progression guidelines should be published.

### Scientific Frontiers and Future Research Trajectories

Keywords represent current research issues or topical concepts, while emerging trends and research frontiers are represented by burst keywords. We used the CiteSpace program to gather burst keywords and identified 2 scientific frontier areas with the strongest citation bursts—video games (2017-2021) and young adults (2018-2021).

#### Video Games

We found that VR video games are simple and appealing to patients and therefore encourage patient participation. Measurements and assessments that were taken after testing VR video games with 12 older patients and 13 children with intellectual disabilities showed clinical improvements in motor capabilities and a positive effect on quality of life [49,50]. Jung et al [51] investigated the impact of Xbox Kinect (Microsoft Corporation) training on lower extremity motor function in adolescents with spastic diplegia cerebral palsy. The outcome measurements, which were measured by using standard industry tools, showed that adolescents displayed significant improvements. A previous randomized controlled trial assessed the impact of a VR dance training program, which involved using an Xbox 360 Kinect game, on kyphosis angle and respiratory parameters in young women with postural hyperkyphosis and showed that dance games were an effective therapy [52]. Deutsch et al [53] asked 15 individuals with mild to moderate lower extremity deficits in the chronic poststroke phase to use the Kinect Light Race game and showed that video games provided comparable intensity, improved accuracy, greater enjoyment, and less exertion when compared with standard care activities. The application of VR-based video games in the field of rehabilitation is the current potential research direction.

#### Young Adults

VR rehabilitation training research has primarily focused on sport rehabilitation aspects, such as the spine, gait, or balance, with fewer studies on teenagers when compared to studies on adults. The burst keyword *young adult* appeared between 2018 and 2021, indicating that scholars have begun to pay attention to the positive physiological, social, and psychological impacts on teenagers. Fralish et al [54], in a case study of 6 VR sessions over a 3-week period, reported that specific VR programs were putatively physiologically and psychosocially helpful (mood improvement) for young adults with physical disabilities. Smith et al [55] proposed a VR job interview training system for participants with severe mental illness and transition-aged youth with autism spectrum disorders, which resulted in improved interview skills and employment access.

### Conclusions

In this study, we used a bibliometric analysis to objectively, comprehensively, and systematically analyze the VR rehabilitation research literature. We identified the knowledge bases, current topical hot spots, and oncoming trends in this area. In this research field, the areas with acceptable knowledge bases included kinematics, neurorehabilitation, brain injury, exergaming, aging, motor rehabilitation, mobility, cerebral palsy, and exercise intensity. We theorize that future emerging trends and frontiers will focus on video games and young adults. We also identified contemporary research topics and future trends in VR rehabilitation research and provided guidance for future research in this exciting area.

Although papers that were published at different times were gathered for this study, some were not comprehensive in nature and may have introduced publication bias, thereby affecting study outcomes.

### Limitations

Our study still has some limitations to be addressed. Although the WSCC database is reliable, other databases, such as non-English databases, should also be considered to ensure that all relevant papers are collected. Additionally, the rapid progress in the field of VR technology application limits the timeliness of this bibliometric study. Further, the VR-based rehabilitation activities we investigated included some gaming exercises, exergames, and video games that were not clearly defined. Finally, the results of the included studies only reflect current research trends in academia. In the future, VR technology is expected to be further integrated with clinical applications, which will enrich and improve the research in this subject field.

## Abbreviations

SCIE: Science Citation Index Expanded
VR: virtual reality
WSCC: Web of Science Core Collection
